# Preoperative Outcome Predictors in Aortic Valve Replacement: A Single-Center Retrospective Study

**DOI:** 10.3390/jcm14155196

**Published:** 2025-07-22

**Authors:** Ilenia Foffa, Augusto Esposito, Ludovica Simonini, Roberta Lombardi, Maria Serena Parri, Angelo Monteleone, Pier Andrea Farneti, Cecilia Vecoli

**Affiliations:** 1 Institute of Clinical Physiology, National Research Council, 54100 Massa, Italy; ilenia.foffa@cnr.it (I.F.); simoniniludovica@gmail.com (L.S.); cecilia.vecoli@cnr.it (C.V.); 2 Fondazione Toscana Gabriele Monasterio, 54100 Massa, Italy; rlombardi@monasterio.it (R.L.); parri@ftgm.it (M.S.P.); 3 Cardiology Unit, Ospedale del Cuore, Fondazione Toscana Gabriele Monasterio, 54100 Massa, Italy; 4 Department of Radiology, Fondazione Toscana Gabriele Monasterio, 54100 Massa, Italy; monteleone@ftgm.it; 5 Adult Cardiac Surgery Division, Ospedale del Cuore, Fondazione Toscana Gabriele Monasterio, 54100 Massa, Italy; farneti@ftgm.it

**Keywords:** aortic valve replacement, outcome predictors, serum albumin ratio (BAR), inflammatory prognostic index (IPI)

## Abstract

**Background**: Several blood biomarkers have shown a major role in predicting major adverse complications (MACs) in patients who have undergone cardiac surgery. Here, we aimed to investigate the possible role of the blood urea nitrogen (BUN) to serum albumin ratio (BAR) and the inflammatory prognostic index (IPI) in predicting major adverse complication after surgical aorta valve replacement (SAVR). **Methods**: The clinical, echocardiographic, and clinical-chemistry laboratory data of 195 patients who underwent SAVR were evaluated. The post-surgical MACs (death, surgical re-exploration, myocardial infarction and cerebral ischemia) during the hospitalization were recorded. Univariate and multivariate logistic regression analyses were studied by comparing the basic clinical features, echocardiographic parameters, and patients’ hematological indices between patients with or without MACs. **Results**: The mean age was 66.1 years, and 62.5% were males. Logistic regression analysis showed that the left atrium volume (LAV), BAR, and IPI as either continuous or categorical variables were independently associated with MACs. Moreover, we found a combined effect of higher LAV with a higher value of BAR or IPI. Combined higher levels of LAV and BAR increased the risk of developing MACs by 9.8 (CI 95% = 2.8–34.3, *p* = 0.0003), while higher values of LAV and IPI increased the risk of developing MACs by 4.5. **Conclusions**: Higher levels of BAR and IPI, alone or in combination with higher LAVs, showed an independent predictive value of MACs after SAVR. These findings strongly support the importance of evaluating easily available biomarkers of the pre-operative status of patients in order to predict adverse outcomes.

## 1. Introduction

The growing global burden of cardiovascular disease, especially among the aging population, has contributed to a rise in high-risk cardiac surgeries [1] such as aortic valve replacement (AVR). Indeed, in many cases, surgical AVR (SAVR) remains the treatment of choice, although open-heart operations still carry an elevated risk of mortality and morbidity [2,3]. Moreover, despite the advancement in therapeutics, reducing major post-operative complications after cardiac surgery remains challenging [3,4,5].

Numerous preoperative risk stratification scores have been created, although more limitations remain in their predictive accuracy toward adverse post-operative outcomes [6,7]. An emerging field of research aims to enrich existing clinical risk prediction models with hematologic parameters [7], since these biomarkers are emerging as optimal predictors of post-surgery outcome in several clinical conditions including cardiovascular disease [8]. The major advantage of blood biomarkers is their ease of collection in daily clinical practice. A defined biochemical signature associated with an adverse post-operative outcome may aid in identifying patients who are at high risk, helping physicians to tailor peri-operative management to reduce later complications.

Among all, the blood urea nitrogen (BUN) to serum albumin ratio (BAR) [9] and the inflammatory prognostic index [10] are two novel indices strongly related to the nutritional and immune-inflammatory status of patients. Both markers have as their denominator albumin, which is the most abundant serum protein with numerous functions: from its well-known role as a plasma-expander to antioxidant, anti-inflammatory, anticoagulant and anti-platelet aggregation activity [11].

Both BAR and IPI have been shown to provide important information about prognosis in patients with cardiovascular disease, and their high levels are associated with poor outcomes [9,10,12,13]. To the best of our knowledge, there is no study in the literature showing the role of BAR and IPI in predicting the risk of new-onset major adverse complications (MACs) in patients after SAVR. Therefore, in this paper, we aimed to investigate the possible role of BAR and IPI in predicting major adverse complications after SAVR.

## 2. Methods

### 2.1. Study Design and Patients

This is a retrospective, observational, and single-center study conducted at Fondazione Toscana G. Monasterio. Between July 2024 and February 2025, 194 consecutive patients undergoing aortic valve replacement (AVR), for severe stenosis or insufficiency, associated or not with thoracic aortic aneurysm, were recruited for this study. This study received ethical approval by the local Ethics Committee (Comitato Etico di Area Vasta Nord Ovest (CEAVNO) Study ID n. 26429/2024 on 11 June 2024). All participants included in the study signed a written informed consent.

All patients underwent routine evaluation before cardiac surgery. This includes a minimum of medical history assessment, clinical examination, electrocardiography, laboratory blood sampling, and echocardiography performed by cardiologists specifically dedicated to cardiac imaging and anesthetic evaluation. Patients with acute endocarditis, experiencing emergent surgeries, or undergoing redo operations were excluded. Regarding echocardiographic parameters, the left atrium (LA) volume was measured using the 4-chamber single-plane Simpson’s method. The left atrium volume index (LAVI) was calculated as LA volume divided by body surface area. The blood urea nitrogen (BUN) to serum albumin ratio (BAR) and the inflammatory prognostic index (IPI) were calculated as previously described [9,10,12,13]. For all patients, severities of aortic valvular dysfunction, types of surgery (full sternotomy, hemi-sternotomy, mini-thoracotomies), and concomitant surgery such as aorta surgery, mitral valve surgery, coronary artery bypass graft (CABG), or left atrial appendage closure (LAAC) for atrial fibrillation were reviewed. The length of hospital stay was calculated in each patient from the day of valvular operation to discharge. The post-surgical complications during the hospitalization were recorded. Major adverse complications (MACs) were considered death, surgical re-exploration, myocardial infarction and cerebral ischemia.

### 2.2. Statistical Analysis

Continuous variables are presented as mean ± standard deviation (SD), while categorical variables are presented as relative percentages. Continuous variables were compared using Student’s *t* test or the Mann–Whitney U test for data with a normal or non-normal distribution, respectively. Categorical variables were compared using the chi-square test. Categorical variables were expressed as percentages, and continuous variables were expressed as mean and standard deviation (SD). Logistic regression analysis was performed to explore the association between the single covariates and risk of developing MACs. In multivariate analyses, BAR and IPI were separately evaluated to avoid collinearity, since they share albumin as a denominator. The LAV, BAR, and IPI values were categorized according to their higher tertile (T3) levels (as higher LAV or BAR or IPI ≥ T3 value) or their lower tertiles (T2 and T1) levels (as lower LAV or BAR or IPI < T2 value). All statistical analyses were completed using Stata/SE 13.1 and SPSS Version 24. A *p*-value < 0.05 was considered statistically significant in this study.

## 3. Results

The general baseline characteristics of the population are described in Table 1.

The mean age was 66.1 years, and 62.5% were males. Bicuspid aortic valve was surgically identified in 43.5% of patients. One hundred and ten (56.5%) patients underwent only aortic valve replacement, while other patients experienced other concomitant surgeries such as aorta surgery, mitral valve surgery, CABG, and LAAC (in 23.2%, 4.6% and 1.5% patients, respectively). Full sternotomy was performed in 29.3% of patients, mini-thoracotomy was performed in 19%, while mini-sternotomy was achieved in 51.7% of patients. Regarding the post-operative outcome, twenty-five patients (12.8%) developed major adverse complications.

In Table 2, the population was stratified according to the development of MACs (MAC group) or not (noMAC group).

Concerning the clinical characteristics, there was no difference between patients of the MAC group compared to the noMAC group except for the type of valve. Patients with major adverse complications were those who underwent a full sternotomy (*p* = 0.01). Patients of the MAC group had a longer hospital stay (*p* < 0.0001), which was most likely for the onset of adverse post-operative complications. No statistically significant differences in cardiovascular risk factors prevalence and treatments were observed between groups.

The pre-operative echocardiographic study showed that patients who developed major adverse complications had a statistically significant higher volume of left atrium index (LAVI, *p* = 0.004), while no differences in other echocardiographic parameters were observed.

Bio-humoral analyses showed that urea (*p* = 0.04), BAR (*p* = 0.02), and the IPI (*p* = 0.01) index were significantly different between the MAC and noMAC groups. Moreover, although albumin is a common denominator of the two indexes, it did not affect either of the two parameters. Indeed, BAR seems to be affected by BUN (*p* = 0.04 between MAC and noMAC groups), while IPI seems to be affected by the C-reactive protein, although the difference between MACs and noMACs did not reach the statistical significance (Figure S1).

**Figure 1:**
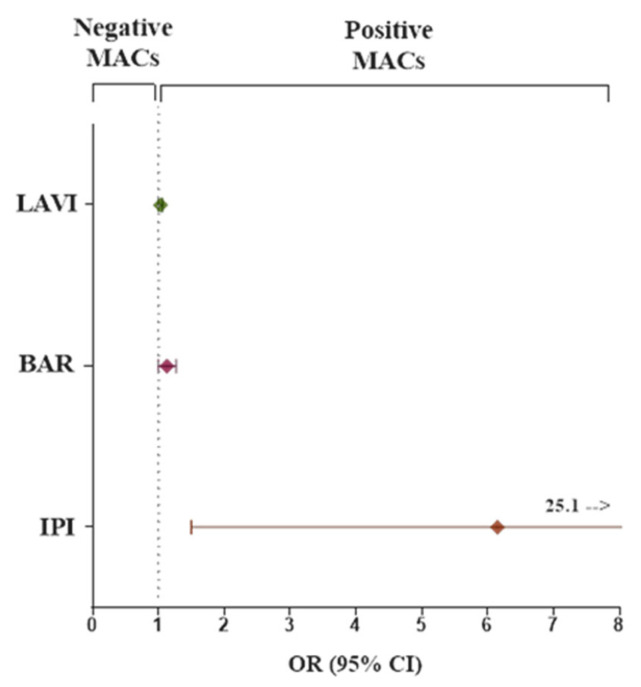
Forest plot of the odds ratio and its 95% confidence interval for comparison of categorical predictor variables (LAVI, BAR, IPI) for the odds of having major adverse complications (MACs). Abbreviations as in Table 3.

Univariate and multivariate logistic regression analyses were performed to identify independent variables determining the development of major adverse complications (Table 3; Figure 1).

The left atrium volume index (LAVI), BAR, and IPI (all as continuous variables) were found to be independently associated with MACs in both univariate and multivariate logistic regression analysis. The results of multivariate analysis did not change also after adjustment for sex and age.

Moreover, when the LAVI, IPI and BAR values were stratified into tertiles (T1, T2, and T3), the highest tertile (T3) of each parameter showed a good accuracy and specificity (67.3 and 69.1; 65.3 and 67.8; 68.4 and 69, respectively) and exhibited a significantly elevated risk of MACs compared to the lowest tertiles (T1 and T2). We further explored the combined value of a higher LAVI with a higher value of hematological biomarkers (higher LAVI + higher BAR or higher LAVI + higher IPI) versus the lower values (lower LAVI + lower BAR or lower LAVI + lower IPI) on risk of MACs. We found that the combined effect of LAVI and BAR increased the risk of developing MACs by 9.8 (CI 95% = 2.8–34.3, *p* = 0.0003), while patients with higher values of LAVI and higher values of IPI had significantly more than a 4.5-fold higher risk for the MACs (CI 95% = 1.3–16.5, *p* = 0.02) (Table 4).

## 4. Discussion

**Figure 2:**
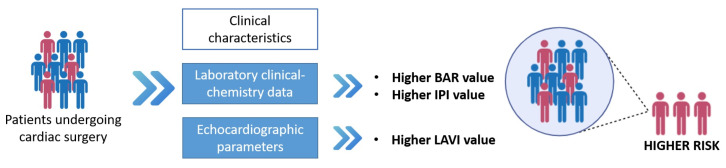
A diagram of the study flow: biomarkers and risk stratification for major adverse complications (MACs).

The main findings of this paper were that BAR and IPI, two emerging parameters of nutritional and immune-inflammatory status, were significantly related to adverse outcome after cardiac surgery in patients with aortic valve disease. This result suggest that these two indices deserve specific attention in clinical practice. Moreover, we also found that pre-operative higher LAVI values were independent risk factors for MACs after SAVR. Notably, combining the echocardiographic parameter with one of two blood biomarkers, we found that patients with higher values of LAVIs and BAR or higher values of LAVIs and IPI had the higher risk of major post-surgical complications (Figure 2).

Aortic valve disease is the third most frequent cardiovascular disease [14]. No effective medical therapy is available so far, and in many patients, SAVR is the only feasible treatment. Unfortunately, despite advancements in surgery and therapeutics, reducing post-operative complications and mortality after cardiac surgery remains challenging, and some patients remain with a poor prognosis even after SAVR. In this study, we aim to evaluate whether the analysis of nutritional and/or immuno- inflammatory pre-operative status may help clinicians in early tailoring and targeting intervention to prevent the onset of post-surgical complications.

In our cohort of patients who underwent SAVR, 12.8% had major adverse outcome. No significant differences in clinical or demographics characteristics were found between patients who did or did not develop MACs. In this population, we firstly showed a statistically significant difference of BAR, IPI, and urea that were higher in those who developed MACs compared with patients without MACs. Lastly, only BAR and IPI were independent predictors of poor peri-operative outcomes.

The blood urea nitrogen to serum albumin ratio is a novel and easily accessible prognostic biomarker that integrates indicators of malnutrition, inflammation, protein metabolism, and renal function. As such, it serves as an effective tool for predicting the prognosis of critically ill patients [9,15].

Previous studies have found that BAR exhibits good mortality prediction in different diseases including acute kidney disease, sepsis, chronic obstructive pulmonary disease, and chronic heart failure [12,16,17,18]. Currently, BAR has been shown to correlate with worse prognosis in patients following cardiac surgery, offering predictive insights about in-hospital mortality [9].

Elevated BAR values reflect an imbalance characterized by increased blood urea nitrogen (BUN) levels and decreased serum albumin levels. Notably, both BUN and serum albumin levels have been independently linked to adverse outcomes in patients with cardiovascular disease [9].

The concentration of blood urea nitrogen can be affected by various factors such as protein intake and consumption, blood volume status, urea excretion, and reabsorption in the renal tubules. Elevated BUN levels have been associated with low cardiac output and activation of the renin–angiotensin–aldosterone system (RAAS) [19,20]. Patients undergoing cardiac surgery frequently experience reduced systemic perfusion that triggers the activation of both the sympathetic nervous system and the RAAS along with the secretion of arginine vasopressin (AVP). The stimulation of these neurohormonal pathways enhances renal water and sodium retention, which in turn increases passive urea reabsorption in the tubules. AVP also induces an overexpression of urea transporters in the final portion of the nephron, further facilitating urea reabsorption and contributing to elevated blood urea levels [20].

The inflammatory prognostic index, a novel marker calculated by the neutrophil/lymphocyte ratio (NLR) multiplied by the C-reactive protein/albumin ratio, has a major prognostic value in oncological patients, and its high levels are associated with poor outcomes [21,22,23]. Several studies are emerging and are reporting a major role also in patients with cardiovascular disease. IPI showed a predictive value in patients undergoing coronary angiography and/or percutaneous coronary intervention [24], in patients with heart failure [25], and in patients with acute myocardial infarction [13]. Our findings revealed that IPI and BAR were associated with a higher risk of developing peri-operative major complications in patients with aortic valve disease undergoing SAVR, suggesting that the evaluation of baseline inflammatory status could be a simple but advantageous method for the risk stratification and disease management of these patients.

Albumin, the common denominator of IPI and BAR, is the most abundant protein in human serum with numerous physiological functions, from the role as a plasma-expander to antioxidant, anti-inflammatory, anticoagulant and anti-platelet aggregation activity. A clear association has been observed between serum albumin levels and all-cause mortality in elderly individuals [26]. Furthermore, several studies have identified serum albumin as a reliable predictor of surgical outcomes [27,28].

The synthesis of albumin occurs in the liver and is influenced by several factors, including diet and nutrition and disease states [29]. Inflammatory states and in particular, high concentrations of the cytokines IL-6 and TNF-alpha, were two of the main factors causing low levels of serum albumin [30]. On the other hand, serum albumin has a pivotal role as circulating anti-inflammatory factor.

Lastly, we found the pre-operative value of LAVI to be independently associated with the onset of MACs in patients who underwent SAVR. The enlargement of the left atrium has been previously reported as a predictor of death, incident heart failure, atrial fibrillation, and stroke in the general population [31]. Moreover, LAV and left ventricular longitudinal strain (LVLS) had a significant prognostic value for mortality and major cardiovascular events in patients with aortic valve disease [32,33,34]. Left atrial dilation is a morphologic sign associated with LV remodeling, longstanding diastolic dysfunction, more advanced disease in AS and an increased risk of progression in patients with severe asymptomatic AS [35]. More recent, Butcher et al. demonstrated the prognostic value of LAV in patients with bicuspid aortic valve and moderate to severe aortic regurgitation [36]. Thus, in line with this evidence, the association between LA enlargement and outcomes after cardiac surgery might become an important critical clinical risk identifier.

We have to recognize several limitations in this study. First, as an observational study, the presence of uncontrolled confounding factors or selection biases cannot be entirely excluded. Second, the research was conducted at a single institution, which may limit the generalizability of the findings to broader populations. Third, the relatively small sample size may have constrained the ability to detect certain statistical associations that could emerge in larger cohorts. Moreover, the heterogeneity of outcomes may have introduced noise in the dependent variable, limiting the interpretability of the predictors. Finally, the possibility of residual or unmeasured confounding factors cannot be fully ruled out.

Despite these limits, the major strength of this study is the identification of easily accessible, inexpensive, and non-invasive biomarkers able to identify patients at increased risk of major adverse complications after surgical procedures.

Moreover, our findings suggest the utility of an integrated use of clinical and hematological biomarkers to ameliorate the preoperative risk stratification.

Current risk stratification scores widely used for patients undergoing heart surgery such as EUROSCORE II and the STS Short-Term Risk Calculator are detailed and provide a reliable framework for risk assessment, but their designs include medical and physiological factors and area under the receiver operating curve values of 0.76 for the STS score and 0.77 for EuroSCORE II for predicting in-hospital mortality [37]. This shows that there is still room for improvement. So, the inclusion in these risk scores of additional parameters regarding nutritional and inflammation status may improve their ability to predict adverse outcomes. Moreover, it could be useful to include in the tradition risk score also other parameters that take into consideration inflammation, nutritional and frailty status but also non-physiological factors such as emotional, behavioral, social and functional factors that are predictive of poor outcomes following cardiac surgery [38]. So, these findings might be integrated into pre-operative workflows, possibly as part of a multimodal score improving performance in predicting mortality or major adverse complications for individual patients undergoing cardiac surgery. In fact, the evidence of altered pre-operative nutritional or immune-inflammatory status may allow clinicians to adopt personalized treatments to improve the health condition of the patient before the surgery. Pre-operative therapeutics adjustments could help to prevent or (early) control the post-operative complications frequently associated with prolonged hospitalization, readmissions and short- and long-term morbidity and mortality and therefore the higher healthcare costs related.

## 5. Conclusions

Our study revealed that IPI and BAR together with LAV are independent predictive risk factors for MACs developed after elective SAVR. Identifying preoperative biomarkers that are efficient in predicting post-operative major complications after SAVR could help develop more effective strategies to obtain a tailored perioperative management. In particular, future prospective studies are needed to evaluate whether interventions able to recover nutritional and immune-inflammatory status can improve the outcome of patients with higher risk of adverse outcomes. Moreover, to support our findings and explore the underlying mechanisms, further better structured trials with higher levels of patient participation are required.

## Figures and Tables

**Table 1:** General baseline characteristics of the study population.

Characteristics	Cohort (*n* = 195)
Age (years) mean ± SD	66.1 ± 11.6
Male (%)	62.5
BMI (kg/m^2^) ± SD	26.9 ± 4.2
Smoking (%)	16
Hypertension (%)	63.5
Dyslipidemia (%)	44.6
Diabetes mellitus (%)	17.9
Hospital stay, day, mean ± SD	8.5 ± 4
Valve disease	
Aortic stenosis (%)	64.6
Aortic regurgitation (%)	35.4
Bicuspid valve (%)	43.5
Therapy	
Anti-hyperlipidemic (%)	41.5
Anti-hypertensive (%)	73.3
Antiplatelet (%)	33.3
Anticoagulant (%)	7.2
Antidiabetic drugs	17.9
Type of surgery	
AVR (%)	56.5
AVR and CABG (%)	7.3
AVR and Aorta surgery (%)	23.2
Aortic, mitral valve surgery (%)	4.6
Aortic, mitral and aorta valve surgery (%)	2.7
AVR and LAAC (%)	1.5
AVR, Aorta, LAAC (%)	1.05
AVR, Aorta, CABG (%)	0.6
Aortic, mitral valve and LAAC (%)	1.5
Aortic, mitral valve and CABG (%)	1.05
Surgical incisions	
Full sternotomy (%)	29.3
Mini-sternotomy (%)	51.7
Mini-thoracotomies (%)	19
Major adverse complications	
Surgical re-exploration *n*, (%)	23 (11.8)
Cerebral ischemia *n*, (%)	1 (0.5)
Death *n*, (%)	1 (0.5)

BMI: body mass index; AVR: aortic valve replacement; LAAC: left atrial appendage closure; CABG: coronary artery bypass graft.

**Table 2:** Comparison of clinical and demographic characteristics, risk factors, echocardiographic parameters, and laboratory clinical-chemistry data among patient groups defined by the onset of major adverse complications.

Characteristics	MAC Group (*n* = 25)	noMAC Group (*n* = 170)	*p* Value
**Clinical characteristics**			
Age (years) mean ± SD	67.7 ± 11.6	65.8 ± 11.6	ns
Male (%)	60	63.5	ns
BMI (kg/m^2^) ± SD	26.8 ± 5.1	26.9 ± 4.1	ns
Smoking (%)	15.3	24	ns
Hypertension (%)	52	65.3	ns
Dyslipidemia (%)	48	44.1	ns
Diabetes mellitus (%)	20	17.6	ns
Atrial fibrillation (%)	12	7.6	ns
Post-operative atrial fibrillation (%)	44	35.3	ns
Hospital stay (day), mean ± SD	16.3 ± 7	9.5 ± 3.3	<0.0001
**Valve disease**			
Aortic stenosis (%)	60	65.3	ns
Aortic regurgitation (%)	40	34.7	ns
**Type of valve**			
Bicuspid valve (%)	28	47	0.04
Tricuspid valve (%)	72	53	0.04
**Therapy**			
Anti-hyperlipidemic (%)	40	42.1	ns
Anti-hypertensive (%)	72	74	ns
Antiplatelet (%)	36	32.9	ns
Anticoagulant (%)	12	5.9	ns
Antidiabetic drugs(%)	16	18.3	ns
**Type of surgery**			
AVR (%)	44	58	ns
AVR and CABG (%)	8	7	ns
AVR and aorta surgery (%)	16	24	ns
Aortic, mitral valve surgery (%)	16	3	ns
Aortic, mitral valve and aorta surgery (%)	8	2	ns
AVR and LAAC (%)	0	2	ns
AVR, aorta, LAAC	0	1	ns
AVR, aorta, CABG	0	1	ns
Aortic, mitral valve and LAAC	4	1	ns
Aortic, mitral valve and CABG	4	1	ns
**Surgical incisions**			
Full sternotomy (%)	52	25.9	0.01
Mini-sternotomy (%)	24	55.3	0.003
Mini-thoracotomies (%)	24	18.2	ns
**Echocardiographic parameters**			
Aorta diameter (mm), mean ± SD	36.20 ± 9.8	39.1 ± 8.1	ns
Aortic root diameter (mm), mean ± SD	35.2 ± 9.3	35.8 ± 6.1	ns
Left atrium volume (mL), mean ± SD	47.3 ± 29.2	36.6 ± 13.4	0.004
Left atrium volume index (LAVI) (mL/m^2^) mean ± SD	26.1 ± 20.4	19.5 ± 7.4	0.004
Left atrium area (cm^2^), mean ± SD	24.8 ± 5.9	22.3 ± 8.2	ns
Left ventricular end-systolic dimension (mm), mean ± SD	51.3 ± 7.4	52.9 ± 11.8	ns
Left ventricular end-systolic volume (mL), mean ± SD	131.2 ± 48.4	134.9 ± 57.6	ns
Ejection fraction (%), mean ± SD	61.4 ± 6.9	60.8 ± 8.5	ns
Peak velocity (m/sec), mean ± SD	3.56 ± 1.24	3.55 ± 1.26	ns
Mean gradient (mmHg), mean ± SD	42.5 ± 21.9	40.9 ± 21.7	ns
Posterior wall thickness (mm), mean ± SD	10.5 ± 2.3	10.8 ± 7.8	ns
Interventricular septum thickness (mm), mean ± SD	12.5 ± 2.9	12.2 ± 2.1	ns
**Laboratory clinical-chemistry data**			
Hemoglobin (Hb), g/dL	13.6 ± 1.8	13.8 ± 1.5	ns
Erythrocytes, ×10^6^/μL	4.6 ± 0.5	4.7 ± 0.6	ns
Neutrophils, ×10^3^/μL	4.15 ± 1.34	4.1 ± 1.46	ns
Lymphocytes, ×10^3^/μL	1.89 ± 0.7	1.85 ± 0.5	ns
Monocytes, ×10^3^/μL	0.61 ± 0.17	0.61 ± 0.6	ns
Platelets, ×10^3^/μL	204 ± 58.8	226 ± 64.2	ns
Erythrocyte sedimentary rate, mm/h	10.2 ± 11.7	10.8 ± 9.1	ns
C-reactive protein (CRP), mg/dL	0.37 ± 0.4	0.28 ± 0.4	ns
Fibrinogen, mg/dL	346.4 ± 115.2	349.8 ± 90.5	ns
Glucose mg/dL	95.7 ± 15	101.6 ± 19	ns
Total cholesterol, mg/dL	162.7 ± 38.2	176.8 ± 38.8	ns
Low-density lipoprotein cholesterol, mg/dL	91 ± 34.6	102 ± 35.2	ns
High-density lipoprotein cholesterol, mg/dL	53.4 ± 15.1	55.2 ± 15.6	ns
Triglycerides, mg/dL	91.2 ± 43.2	101.3 ± 49.2	ns
Lipoprotein(a), nmol/L	51.3 ± 70.5	54.1 ± 66.7	ns
Creatinine, mg/dL	0.99 ± 0.2	0.92 ± 0.5	ns
Creatine phosphokinase, IU/L	106.7 ± 65.1	110 ± 80.5	ns
Alanine aminotransferase, IU/L	22.7 ± 18.9	20.6 ± 15.3	ns
Gamma-glutamyl transferase, IU/L	37.2 ± 41	42 ± 81	ns
Thyroid-stimulating hormone, mU/L	5.8 ± 15	7.4 ± 60	ns
Albumin, g/dL	4.2 ± 0.3	4.3 ± 0.2	ns
Urea mg/dL	45.6 ± 13.9	39.8 ± 12.8	0.04
BAR, mean ± SD	10.8 ±3.7	8.8 ± 3.8	0.02
IPI, mean ± SD	0.28 ± 0.4	0.14 ± 0.2	0.01

List of abbreviation: BMI: body mass index; AVR: aortic valve replacement; LAAC: left atrial appendage closure; CABG: coronary artery bypass graft; IPI: inflammatory prognosis index; BAR: blood urea nitrogen to albumin ratio.

**Table 3:** Univariate and multivariate logistic regression analyses showing the independent risk factors for major adverse complications after aortic valve replacement.

Variables	OR	95% Confidence Interval	*p*-Value
**Univariate analysis**			
LAVI	1.03	1.005–1.05	0.01
BAR	1.1	1.0–1.2	0.03
IPI	4.06	1.15–14.2	0.02
Multivariate analysis *			
LAVI	1.031	1.001–1.062	0.04
BAR	1.13	1–1.27	0.04
IPI	6.15	1.5–25.1	0.01

List of abbreviation: IPI: inflammatory prognosis index; BAR: blood urea nitrogen to albumin ratio; LAVI left atrium volume indexed. * Adjusted for sex and age.

**Table 4:** Odds ratio of the highest tertile of each parameter and odds ratio of combined echocardiographic and blood biomarkers for MACs.

Variables	OR	95% Confidence Interval	*p*-Value
higher LAVI	2.5	1.1–5.9	0.04
higher IPI	1.7	1.0–4.3	0.05
higher BAR	3.7	1.5–9.0	0.004
higher LAVI + higher BAR	9.8	2.8–34.3	0.0003
higher LAVI + higher IPI	4.5	1.3–16.5	0.02

List of abbreviation: IPI: inflammatory prognosis index; BAR: blood urea nitrogen to albumin ratio; LAVI: left atrium volume indexed; MACs: major adverse complications.

## Data Availability

Data available on request due to restrictions (e.g., privacy, legal or ethical reasons).

## Supplementary Materials

The following supporting information can be downloaded at https://www.mdpi.com/article/10.3390/jcm14155196/s1, Figure S1: The behavior of each component of BAR and IPI index across the groups.
