# Supplementary figures and images for: Preoperative Outcome Predictors in Aortic Valve Replacement: A Single-Center Retrospective Study
Source: J Clin Med. 2025 Jul 22;14(15):5196. doi: 10.3390/jcm14155196 (PMC12346984; doi:10.3390/jcm14155196)

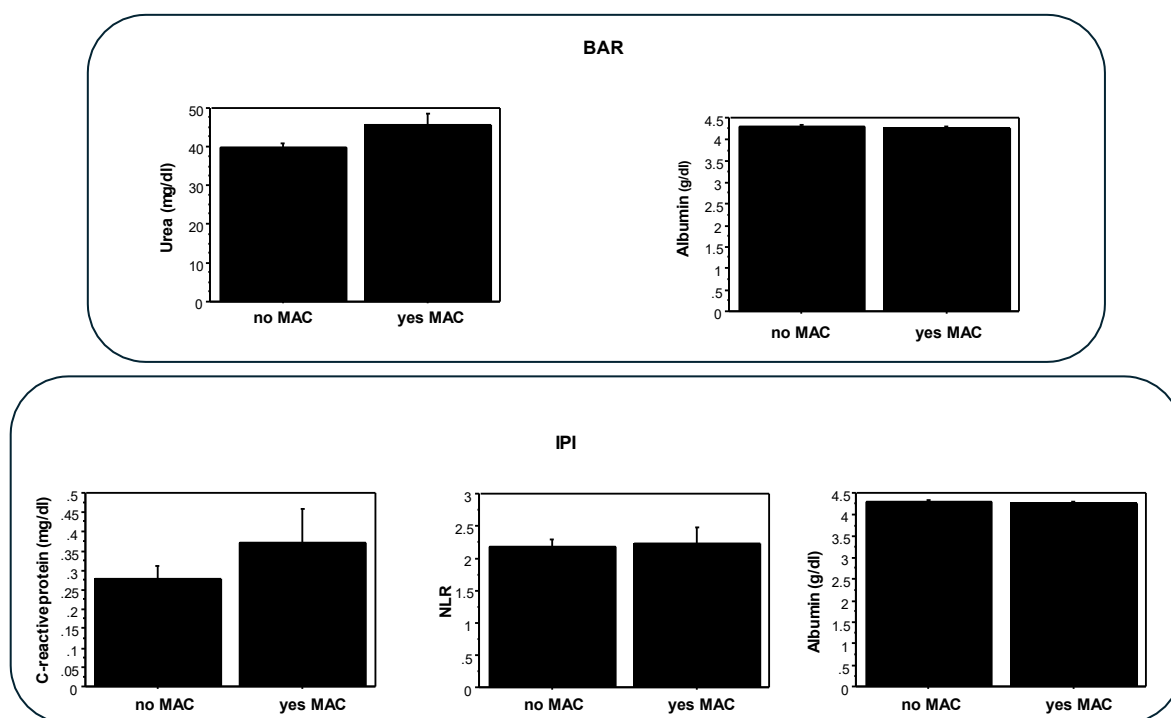

**Figure S1.** The behaviour of each component of BAR and IPI index across the groups

Supplement: Supplementary file 1 [file jcm-14-05196-s001.zip › jcm-3735000-supplementary.pdf]
